# Phénotypes de résistance des souches d’*Escherichia coli* responsables d’infection urinaire au laboratoire du Centre Hospitalo-Universitaire de Befelatanana Antananarivo

**DOI:** 10.11604/pamj.2017.26.166.11828

**Published:** 2017-03-22

**Authors:** Zafindrasoa Domoina Rakotovao-Ravahatra, Fidiniaina Mamy Randriatsarafara, Saïda Rasoanandrasana, Léa Raverohanta, Andriamiadana Luc Rakotovao

**Affiliations:** 1 Unité Laboratoire du CHU-HJRB d’Antananarivo, Madagascar; 2 Département de Santé Publique Faculté de Médecine d’Antananarivo, Madagascar; 3 Département de Biologie Médicale Faculté de Médecine d’Antananarivo, Madagascar

**Keywords:** Antibiotique, Escherichia coli, phénotype, résistance, Antibiotic, Escherichia coli, phenotype, resistance

## Abstract

**Introduction:**

I’infection urinaire à *Escherichia coli* est fréquente en milieu hospitalier. Cette étude se propose de décrire les différents phénotypes de résistance des souches d’*Escherichia coli* afin de surveiller leur émergence.

**Méthodes:**

Il s’agit d’une étude rétrospective de type descriptif de 102 souches d’*Escherchia coli* responsables d’infection urinaire sur une période allant du mois de Janvier 2014 au mois d’Octobre 2016 au Laboratoire du Centre Hospitalo-Universitaire Befelatanana Antananarivo.

**Résultats:**

La résistance aux béta-lactamines a identifié des pénicillinases de haut niveau 50% (n=51), des *Escherichia coli* sécrétrices de Béta-Lactamase à Spectre Etendu E-BLSE 22,5% (n=23), des céphalosporinases de haut niveau 14,7% (n=15), des pénicillinases de bas niveau 5,9% (n=6), des souches sauvages 5,9% (n=6) et une souche d’*Escherichia coli* hautement résistante émergente. La résistance aux aminosides a concerné 58 (56,9%) phénotypes sauvages, 29 (28,4%) souches sensibles à l’amikacine et 15 (14,7%) résistants à tous les aminosides. La résistance aux fluoroquinolones a identifiée 52 (51%) souches sauvages, 9 (8,8%) souches sensibles à la ciprofloxacine et 41 (40,2%) résistantes à tous les fluoroquinolones. Les femmes (25, 7%) (p= 0,25, NS), les sujets de plus de 60 ans (38,7%) (p=0,02), les sujets hospitalisés dans le service de néphrologie (53,8%) (p=0,04), ayant présenté des troubles urinaires et rénaux (29, 7%) (p= 0,2, NS), ont été les plus affectés par les E-BLSE.

**Conclusion:**

La multi-résistance élevée des souches d’*Escherichia coli* interpelle sur une révision du traitement empirique des infections urinaires.

## Abstract (en)

**Introduction:**

Urinary tract infection caused by Escherichia coli frequently occurs in the hospital environment. This study aims to describe resistant phenotypes of Escherichia coli strains to monitor their occurrence.

**Methods:**

We conducted a descriptive retrospective study of 102 Escherchia coli strains responsible for urinary tract infection in the laboratory of the University Hospital Joseph Raseta Befelatanana, Antananarivo from January 2014 to October 2016.

**Results:**

Beta-lactam antibiotic resistance screening identified high-level penicillinases 50% (n=51), Escherichia coli producing extended-spectrum beta-lactamases (ESBLs) 22.5% (n=23), high-level cephalosporinases 14.7% (n=15), penicillinases low level 5.9% (n=6), wild type strains 5.9% (n=6) and a strain ofEscherichia coli emerging strain high-level resistance. Aminoglycosides resistance was identified in 58 (56.9%) wild type phenotype, 29 (28.4%) strains sensitive to amikacin and 15 (14.7%) resistant to all aminoglycosides. Fluoroquinolones resistance was identified in 52 (51%) wild type strains, 9 (8.8%) strains sensitive to ciprofloxacin and 41 (40.2%) resistant to all fluoroquinolones. Women (25, 7%) (p= 0.25, NS), patients more than 60 years (38.7%) (p=0.02), patients hospitalized in the Department of Nephrology (53.8%) (p=0.04), with urinary disorder and kidney disease (29, 7%) (p= 0.2, NS) were the most affected by E-ESBL.

**Conclusion:**

Based on high multidrug resistance in Escherichia coli strains guidelines for the empirical treatment of urinary tract infections need to be revised.

## Introduction

L’infection urinaire constitue un véritable problème majeur de santé publique. Selon l’Organisation Mondiale de la Santé (OMS), les infections urinaires à *Escherichia coli (E. coli)* sont de loin les plus fréquentes au sein de l’hôpital et de la communauté [1]. Au cours de ces dernières années, une augmentation de l’incidence des résistances aux antibiotiques des germes responsables d’infection urinaire a été constatée. L’émergence des Entérobactéries sécrétrices de Bêta- Lactamase à Spectre Etendu (E-BLSE) est de plus en plus prévalente [2]. A Madagascar, peu de données évaluent la sensibilité des germes responsables des infections urinaires de l’adulte en milieu hospitalier [3]. Les infections urinaires à *E. coli* constituent une priorité en matière de surveillance et d’étude de résistance aux antibiotiques étant donné leur fréquence élevée et leur gravité. D’où l’objectif général de cette étude qui consistait à décrire les différents phénotypes de résistance aux antibiotiques des souches d’*E. coli* responsables d’infection urinaire afin de surveiller leur émergence et limiter leur diffusion au sein de l’hôpital et de la communauté. Les objectifs spécifiques étaient de: (i) Identifier les phénotypes de résistance aux antibiotiques des souches (ii) Décrire les causes de leur émergence (iii) Proposer des suggestions pour limiter la diffusion de ces souches au sein de l’hôpital et de la communauté.

## Méthodes

Il s’agit d’une étude rétrospective de type descriptif sur une période de 2 ans et 10 mois allant du 01 Janvier 2014 au 31 Octobre 2016 à l’Unité Laboratoire du Centre Hospitalo-Universitaire d’Antananarivo Hôpital Joseph Raseta Befelatanana (CHUA-HJRB). Tous les résultats des antibiogrammes des patients atteints d’infection urinaire à *E. coli* ont été exploités. L’Unité Laboratoire du CHUA-HJRB est un laboratoire d’analyses médicales polyvalent. Ainsi, le laboratoire de bactériologie est inclus dans ce laboratoire. L’infection urinaire a été recherchée par l’Examen Cyto-Bactériologique des urines (ECBU). L’ECBU a commencé par la cytologie qui a été appréciée par l’examen direct sur cellule de Mallasez et sur frottis urinaire après coloration de gram. Elle a été suivie par la culture des souches sur URISELECT. Par la suite, les souches d´*E. coli* ont fait l´objet d´une identification biochimique avec le système API 20E (BioMérieux). L’infection urinaire à *E. coli* a constitué le critère de positivité de la présente étude avec une leucocyturie supérieure ou égale à 10^4^/ml et une bactériurie supérieure ou égale à 10^3^ UFC/ml [4]. Finalement, un antibiogramme a été réalisé par la méthode de diffusion sur milieu Mueller Hinton selon les recommandations du Comité de la Société Française de Microbiologie CA-SFM [5]. La lecture interprétative de l´antibiogramme a permis de détecter plusieurs phénotypes de résistance. La saisie et le traitement des données ont été effectués sur le logiciel Epi-info 3.5.2. La comparaison des pourcentages a fait appel aux tests de Chi carré de Mantel Haenszel ou le Chi carré corrigé de Yates en cas de faible effectif. Le seuil de signification statistique utilisé a été de p= 0,05.

## Résultats

**Figure 1:**
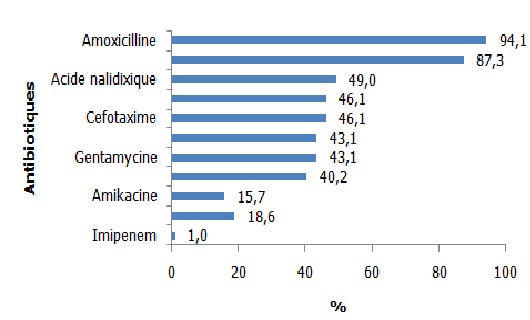
Résistance des souches d’*Escherichia coli* aux antibiotiques

**Figure 2:**
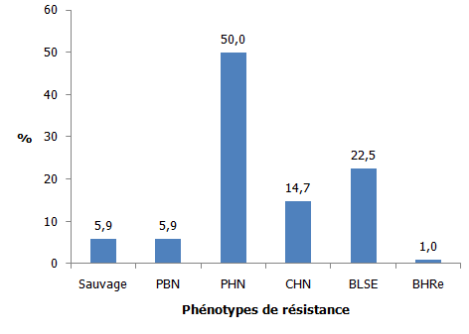
Phénotypes de résistance des souches d’*Escherichia coli* aux bêta-lactamines

**Figure 3:**
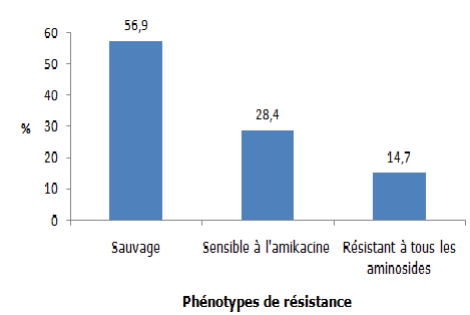
Phénotypes de résistance des souches d’*Escherichia coli* aux aminosides

**Figure 4:**
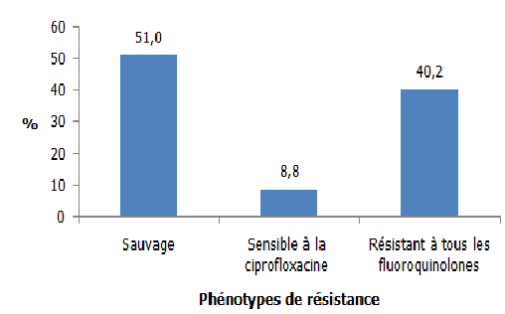
Phénotypes de résistance des souches d’*Escherichia coli* aux fluoroquinolones

Sur une période de 2 ans et 10 mois, on a mis en évidence 102 souches d’*E. coli*. Les femmes, les sujets de 20 à 60 ans ainsi que les sujets hospitalisés en service de maladies infectieuses étaient les plus affectés. La fièvre ainsi que les troubles urinaires et rénaux ont été les signes cliniques les plus observés. La cytologie urinaire a montré des érythrocytes, des infiltrats de polynucléaires neutrophiles, des cellules épithéliales, des levures et des cristaux urinaires dont les cristaux d’oxalate de calcium étaient les plus nombreux. Concernant la résistance aux antibiotiques testés, les résistances à l’amoxicilline 94,1% (n=96) et à l’association Amoxicilline-Acide Clavulanique 87,3% (n=89) étaient de loin les plus fréquentes (Figure 1). La résistance aux béta-lactamines a montré que les pénicillinases de haut niveau (PHN) étaient les phénotypes les plus fréquemment rencontrés 50% (n=51), suivis par les *Escherichia coli* sécrétrices de Bêta-Lactamase à Spectre Etendu dénommées Entérobactéries sécrétrices de Bêta-Lactamase à Spectre Etendu (E-BLSE) 22,5% (n=23) et les céphalosporinases de haut niveau (CHN) 14,7% (n=15). Les pénicillinases de bas niveau (PBN) et les souches sauvages ont été peu représentés avec une fréquence de 5,9% (n=6) chacun. Une seule souche a été résistante à l’Imipenem due à la présence de carbapénèmase. D’où son appellation Bacille Hautement Résistant et Emergent (BHRe) (Figure 2). Concernant la résistance aux aminosides, 58 (56,9%) souches étaient des phénotypes sauvages, 29 (28,4%) étaient des phénotypes sensibles à l’amikacine et 15 (14,7%) souches ont été résistantes à toutes les aminosides (Figure 3). La résistance aux fluoroquinolones a été identifiée avec 52 (51%) souches sauvages, 9 (8,8%) souches sensibles à la ciprofloxacine et 41 (40,2%) souches résistantes à tous les fluoroquinolones (Figure 4). Concernant les souches d’E-BLSE qui représentent les Bactéries Multi-Résistantes (BMR), 3 sur 23 souches ont présenté une résistance associée aux aminosides et 16 sur 23 une résistance associée aux fluoroquinolones. Les femmes (25, 7%) (p= 0,25, NS), les sujets de plus de 60 ans (38,7%) (p=0,02), les sujets hospitalisés dans le service de néphrologie (53,8%) (p=0,04) ayant présenté des troubles urinaires et rénaux (29, 7%) (p= 0,2, NS) ont été les plus affectés par ces E-BLSE (Tableau 1).

**Tableau 1:** Facteurs associés aux souches d’*Escherichia coli* BLSE

Paramètres	*E. coli* BLSE (n=23)		*E. coli* non BLSE (n=79)		
	Effectif	%	Effectif	%	p
**Tranche d'âge**					
0-19ans	1	6,3	15	93,8	0,02
20-59ans	10	18,2	45	81,8	
60ans et plus	12	38,7	19	61,3	
**Genre**					
Masculin	5	15,6	27	84,4	0,25 (NS)
Féminin	18	25,7	52	74,3	
**Service**					
Maladies infectieuses	10	17,5	47	82,5	0,04
Nephrologie	7	53,8	6	46,2	
Externe	2	20,0	8	80,0	
Autres services+	4	18,2	18	81,8	
**Renseignements cliniques**					
Fièvre	8	22,2	28	77,8	0,2 (NS)
Troubles urinaires et rénaux++	11	29,7	26	70,3	
Diabète	1	9,1	10	90,9	
Autres	3	16,7	15	83,3	

+ Hépato-gastro-entérologie, pneumologie, rhumato-dermatologie, cardiologie, neuropsychiatrie, endocrinologie, pédiatrie.

++ Bandelette urinaire positive, dysurie, pollakiurie, pyurie, oligo-anurie, douleur pelvienne et lombaire, syndrome néphrotique, insuffisance rénale.

## Discussion

La présente étude a mis en évidence plusieurs phénotypes de résistance des souches d’E. coli avec prédominance des résistances de haut niveau et des E-BLSE responsables de multi-résistance.

Concernant les infections urinaires à *E. coli*, la prédominance féminine serait liée à la configuration anatomique : brièveté de l’urètre, proximité des orifices génital et anal, insuffisance des pratiques d’hygiène, rapport sexuel et grossesse [6]. Les sujets entre 20 à 60 ans en période d’activité génitale étaient souvent affectés. Cela peut être expliqué par le fait que leurs activités professionnelles, culturelles et sportives pourraient être des facteurs favorisants chez ces sujets. Les sujets hospitalisés en service de maladies infectieuses, la fièvre ainsi que les troubles urinaires et rénaux ont été les plus observés. Cela démontre que les patients ne viennent en consultation que lorsque l’infection urinaire arrive au stade de gravité. En effet, l’importance du traitement empirique des infections urinaires chez le médecin traitant va favoriser les résistances aux antibiotiques. De même, l’automédication antibiotique se pose avec gravité dans les pays en développement où ces médicaments sont facilement disponibles souvent sans ordonnance médicale [7].

L’étude de la sensibilité des antibiotiques a montré des résistances importantes des souches d’*E. coli* à l’ensemble des antibiotiques testés. La résistance aux aminopénicillines (amoxicilline) est la plus fréquente. Ce résultat est similaire à d’autres études [4, 8, 9]. Cette résistance est acquise et serait la conséquence de la pression de sélection liée à la consommation abusive de ces antibiotiques dans les pays en développement [6]. Ces taux élevés de résistance à l’amoxicilline justifient que les aminopénicillines ne soient plus actuellement recommandées en traitement probabiliste des infections urinaires [4].

Concernant les phénotypes de résistance aux Bêta-lactamines, le mécanisme essentiel de la résistance est de nature enzymatique par production de bêta-lactamase. Aussi a-t-on observé de rares souches de pénicillinases de bas niveau (PBN) qui secrètent une pénicillinase plasmidique dénommée TEM 1, à l’origine de la résistance de bas niveau [10]. Le nom TEM provient du nom du malade chez qui on a isolé la première souche porteuse de ce type d’enzyme. Les souches de pénicillinases de haut niveau (PHN) étaient les plus fréquentes représentant la moitié des cas (50%). Il s’agit d’une haute résistance aux oxapénames concernant l’association amoxicilline - acide clavulanique. Cette résistance pourrait s´expliquer par une baisse de l´activité de l´inhibiteur des bêta-lactamases (acide clavulanique), résultante d´une hyperproduction de pénicillinase, ou de l´inactivation de l´inhibiteur lui-même [6]. Cela est probablement dû à la prescription souvent empirique de cette molécule particulièrement en médecine ambulatoire dans l’attente des résultats des ECBU. Les souches résistantes aux céphalosporines ont été représentées par les E-BLSE suivies des céphalosporinases de haut niveau (CHN) par ordre de fréquence. Les CHN étaient probablement reliées à la sélection en cours de traitement de mutants hyperproducteurs de céphalosporinases chromosomiques chez l’espèce *E. coli* [10]. Quant aux BLSE, elles étaient dérivées des enzymes de type TEM et SHV (pour sulfhydril-variable) dans les années 1990 et diffusaient majoritairement au sein de clones hospitaliers de *Klebsiella pneumoniae* et d’*Enterobacter sp*. Toutefois, la diffusion de CTX-M (Céfotaximase-Munich) au sein de l’espèce E coli a bouleversé cette situation. Les CTX-M constituent désormais la majorité des BLSE quelle que soit la région du monde à tel point qu’on qualifie leur diffusion de pandémie [11]. Les BLSE de type CTX-M ont un plus haut niveau de résistance au céfotaxime (ou ceftriaxone), céfépime et l’aztréonam qu’à la ceftazidine. Certaines d’entre elles ont évolué plus récemment par mutation générant également un haut niveau de résistance à la ceftazidine [10]. Ainsi, Les E-BLSE hydrolysent la majorité des bêta-lactamines en n’épargnant que les céphamycines (comme la céfoxitine) et les carbapénèmes (Imipenem) [11].

Par ailleurs, ces E-BLSE avaient fréquemment des résistances associées aux autres antibiotiques en particulier aux fluoroquinolones. Cela peut être expliqué par le fait que les gènes des E-BLSE, portés généralement par des plasmides, seraient souvent associés à des gènes de résistance aux autres antibiotiques [4]. Ainsi, les E-BLSE sont aujourd’hui les Bactéries Multi-Résistantes (BMR) majoritaires dans de nombreux pays [12]. La fréquence significativement élevée des E-BLSE chez les sujets âgés pourrait être la conséquence de la pression des antibiotiques, de la fragilité de ces patients, de l’utilisation répétée des antibiotiques de la même famille et des infections à répétition [6, 13]. De même, les patients hospitalisés en service de néphrologie étaient significativement les plus touchés et les troubles urinaires et rénaux étaient les plus fréquents tels que la pyurie, la pyélonephrite voire l’insuffisance rénale. En effet, les E-BLSE sont à l’origine d’infections potentiellement sévères et de prescriptions d’antibiotiques à large spectre bactérien. Leur émergence est aussi bien hospitalière que communautaire. De plus, les mesures « habituelles » d’hygiène ne permettent pas de répondre à la propagation des E-BLSE, leur prévalence étant en constante augmentation [12]. Par ailleurs, on a également observé une souche d’Entérobactérie Productrice de Carbapénèmase (EPC) dénommée actuellement Bactérie Hautement Résistante et Emergente (BHRe) [14]. Les BHRe constituent avec les E-BLSE la menace infectieuse la plus sérieuse pour les prochaines années, en ville (E-BLSE) et à l’hôpital (E-BLSE et BHRe). Les infections aux BHRe sont associées à une morbi-mortalité et des coûts élevés et posent des problèmes majeurs de traitement du fait d’un nombre limité d’alternatives thérapeutiques [15]. La stratégie de prévention des BHRe repose sur différents niveaux dont l’application systématique des précautions standard pour tout patient avec une attention particulière sur la gestion des excréta, des précautions complémentaires d’hygiène et des précautions spécifiques pour les patients porteurs de BHRe [14]. De même, le contrôle des E-BLSE passe autant par la réduction de l’utilisation des antibiotiques que par l’interruption de leur transmission manuportée. La réduction des volumes antibiotiques, en ville et à l’hôpital est un objectif impératif et urgent. Du côté des précautions d’hygiène, le respect des précautions standard en toute situation et l’application des précautions contact pour les porteurs d’E-BLSE doivent permettre de limiter la diffusion de ces souches [16].

Concernant les phénotypes de résistance aux aminosides, le mécanisme relève essentiellement de l’acquisition d’enzymes modificatrices. Ces enzymes appartiennent à 3 classes correspondant à des activités de phosphorylation, acétylation et nucléotidylation [17]. Dans la présente étude, la présence des phénotypes résistants à tous les aminosides devrait être prise en compte. En effet, l’émergence actuelle des méthyltransférases de l’ARN 16S conférant un haut niveau de résistance à tous les aminosides utilisés en pratique est un phénomène préoccupant qui nécessite un suivi épidémiologique [17].

Concernant les phénotypes de résistance aux fluoroquinolones, 40,2% ont été résistants à tous les fluoroquinolones. Cette proportion est très élevée par rapport à d’autres études comme celle effectuée au Maroc qui n’a montré que 27% de résistance [18]. Cela témoigne le haut niveau de résistance aux fluoroquinolones dans notre pays. Plusieurs mécanismes pourraient être responsable de cette résistance: 1) une imperméabilité de la paroi bactérienne par réduction de l’expression ou une inactivation du gène codant pour les porines, (2) une mutation de gènes de la région QRDR, codant pour la sous-unité gyrA de l’ADN-gyrase (gènes gyrA/B) ou de la topoisomérase IV (gène parC/E) ou (3) l’acquisition ou la surexpression d’une pompe à efflux réduisant la concentration des fluoroquinolones dans les bactéries. Ces résistances surviennent principalement par mutations successives au niveau des gènes chromosomiques des cibles des quinolones: la première étape mutationnelle (« first step mutation ») témoigne d’un premier niveau de résistance [19]. Il est maintenant établi que la résistance d’*E. coli* aux quinolones est corrélée à la consommation ambulatoire de quinolones, à l’échelle des états, des hôpitaux, des cabinets de médecine générale et de la communauté en général [20]. Le respect strict des recommandations d’antibiothérapie des infections courantes doivent permettre de limiter drastiquement l’utilisation des quinolones dans les infections urinaires, en limitant les indications et les durées du traitement, et en pratiquant une désescalade thérapeutique quand l’antibiogramme le permet. Une modification majeure des pratiques de prescription est rapidement nécessaire, afin de sauvegarder l’efficacité de cette classe thérapeutique très utile en pratique courante.

La présente étude a mis en exergue l’évolution croissante des résistances bactériennes aux antibiotiques qui nécessite des mesures radicales. Ainsi, devant toute suspicion d’infection urinaire, il est préférable de faire un ECBU avec antibiogramme obligatoire. En effet, l’antibiogramme est avant tout un outil d’aide à la décision thérapeutique: en catégorisant la bactérie sensible, intermédiaire ou résistante, il guide avec prédictibilité l’antibiothérapie, contribuant à un gain en morbi-mortalité selon la gravité des infections bactériennes concernées [21]. Cela va éviter le traitement probabiliste ou empirique de l’infection urinaire responsable des résistances. De même, l’automédication devrait être évitée en contrôlant l’approvisionnement des antibiotiques au niveau de la communauté et de l’hôpital. Les précautions d’hygiène standards et particulières devraient également être respectées en cas d’E-BLSE ou BHRe. Une prise de conscience des autorités sanitaires, des professionnels de santé et de la population est nécessaire pour que ces mesures soient comprises et respectées.

## Conclusion

Parmi les souches d’*E. coli* identifiées dans la présente étude, les phénotypes résistantes étaient fréquentes telles que celles qui possédaient des pénicillinases de haut niveau et qui étaient résistantes aux quinolones et aux aminosides. De même, la proportion des E-BLSE-BMR n’étaient pas négligeable. L’importance de ces résistances aux antibiotiques est probablement la conséquence de l’usage inapproprié de ces molécules dans notre pays surtout des pénicillines qui sont utilisés fréquemment au cours de n’importe quelle infection. L’automédication représente également un réel problème favorisant rapidement les émergences et les résistances des germes. Par conséquent, la sensibilisation des médecins à demander un examen cytobactériologique des urines avec antibiogramme est primordiale pour tout patient présentant des signes suspects d’infection urinaire. Il faut prévoir également une bonne stratégie d’approvisionnement et de dispensation des antibiotiques pour éviter l’automédication. Le traitement empirique des infections urinaires dans notre pays devrait également être révisé. De même, le contrôle des E-BLSE passe autant par la réduction de l’utilisation des antibiotiques que par l’interruption de leur transmission manu-portée.

### Etat des connaissances actuelles sur le sujet

- Infection urinaire: les infections urinaires font partie des infections les plus fréquentes en milieu hospitalier;
- Escherichia coli: *Escherichia coli* est de loin le germe le plus fréquemment retrouvé dans les infections urinaires;
- Augmentation des phénotypes de résistance aux antibiotiques: de nombreux phénotypes de résistance des souches d’Escherichia coli aux antibiotiques apparaissent actuellement et sont favorisés par les traitements empiriques, les traitements probabilistes et les automédications.

### Contribution de notre étude à la connaissance

- Mécanisme des phénotypes de résistance: la présente étude décrit de façon détaillée les différents phénotypes de résistance des souches d’Escherichia coli avec leurs mécanismes d’apparition respectifs qui peuvent exister en milieu hospitalier;
- Précautions d’hygiène en cas de BLSE et BHRe: la présente étude met en exergue la nécessité des mesures d’hygiène pour éviter la propagation des Escherichia coli BLSE et BHRe en particulier les précautions standards, les précautions complémentaires d’hygiène et les précautions spécifiques;
- Importance de l’ECBU avec antibiogramme: la présente étude souligne l’importance de l’examen cyto-bactériologique des Urines avec antibiogramme avant tout traitement des infections urinaires pour éviter les traitements empiriques et probabilistes qui peuvent favoriser l’émergence et la résistance des germes aux antibiotiques.
